# The Genotype of the Donor for the (GT)_n_ Polymorphism in the Promoter/Enhancer of *FOXP3* Is Associated with the Development of Severe Acute GVHD but Does Not Affect the GVL Effect after Myeloablative HLA-Identical Allogeneic Stem Cell Transplantation

**DOI:** 10.1371/journal.pone.0140454

**Published:** 2015-10-16

**Authors:** Víctor Noriega, Carolina Martínez-Laperche, Elena Buces, Marjorie Pion, Noemí Sánchez-Hernández, Beatriz Martín-Antonio, Vicent Guillem, Anna Bosch-Vizcaya, Leyre Bento, Milagros González-Rivera, Pascual Balsalobre, Mi Kwon, David Serrano, Jorge Gayoso, Rafael de la Cámara, Salut Brunet, Rafael Rojas-Contreras, José B. Nieto, Carmen Martínez, Marcos Gónzalez, Ildefonso Espigado, Juan C. Vallejo, Antonia Sampol, Antonio Jiménez-Velasco, Alvaro Urbano-Ispizua, Carlos Solano, David Gallardo, José L. Díez-Martín, Ismael Buño, Spanish Hematopoietic Stem Cell Transplantation and Cell Therapy Group (GETH)

**Affiliations:** 1 Department of Hematology, Hospital General Universitario Gregorio Marañón, Madrid, Spain; 2 Instituto de Investigación Sanitaria Gregorio Marañón (IiSGM), Madrid, Spain; 3 Department of Inmunology, Hospital General Universitario Gregorio Marañón, Madrid, Spain; 4 Department of Hematology, Hospital Clinic, University of Barcelona, IDIBAPS, Instituto de Investigación Josep Carreras (IJC), Barcelona, Spain; 5 Department of Hematology and Medical Oncology, Hospital Clínico Universitario de Valencia, Universitat de Valencia, Instituto de Investigación Sanitaria INCLIVA, Valencia, Spain; 6 Department of Hematology, ICO Girona, Hospital Josep Trueta, IDIBGI Foundation, Girona, Spain; 7 DNA Sequencing Core Facility, Hospital General Universitario Gregorio Marañón, Madrid, Spain; 8 Department of Hematology, Hospital La Princesa, Madrid, Spain; 9 Department of Clinical Hematology, Hospital de la Santa Creu i Sant Pau, Barcelona, Spain; 10 Department of Hematology, Hospital Reina Sofia, Cordoba, Spain; 11 Department of Hematology, Hospital Morales Meseguer, Murcia, Spain; 12 Department of Hematology, Hospital Clínic, Barcelona, Spain; 13 Department of Hematology, University Hospital of Salamanca, Salamanca, Spain; 14 Department of Hematology and Hemotherapy, Hospital Universitario Virgen del Rocío, Seville, Spain; 15 Department of Hematology, Hospital Universitario Central de Asturias, Oviedo, Spain; 16 Department of Hematology, Hospital Universitario Son Espases, Palma de Mallorca, Islas Baleares, Spain; 17 Department of Hematology, Hospital Regional Universitario de Málaga, Málaga, Spain; German Red Cross Blood Service Frankfurt, GERMANY

## Abstract

The *FOXP3* gene encodes for a protein (Foxp3) involved in the development and functional activity of regulatory T cells (CD4+/CD25+/Foxp3+), which exert regulatory and suppressive roles over the immune system. After allogeneic stem cell transplantation, regulatory T cells are known to mitigate graft *versus* host disease while probably maintaining a graft *versus* leukemia effect. Short alleles (≤(GT)_15_) for the (GT)_n_ polymorphism in the promoter/enhancer of *FOXP3* are associated with a higher expression of *FOXP3*, and hypothetically with an increase of regulatory T cell activity. This polymorphism has been related to the development of auto- or alloimmune conditions including type 1 diabetes or graft rejection in renal transplant recipients. However, its impact in the allo-transplant setting has not been analyzed. In the present study, which includes 252 myeloablative HLA-identical allo-transplants, multivariate analysis revealed a lower incidence of grade III-IV acute graft *versus* host disease (GVHD) in patients transplanted from donors harboring short alleles (OR = 0.26, CI 0.08–0.82, p = 0.021*);* without affecting chronic GVHD or graft *versus* leukemia effect, since cumulative incidence of relapse, event free survival and overall survival rates are similar in both groups of patients.

## Introduction

Allogeneic stem cell transplantation (allo-SCT) is nowadays the therapy of choice for several neoplastic and non-neoplastic diseases [1]. After allo-SCT, donor derived immunocompetent cells recognize recipient cellularity and promote an immunological reaction called graft *versus* host disease (GVHD), which is one of the most important causes of morbi-mortality after allo-SCT [2]. However, donor *versus* recipient immune reactions also harbor a beneficial effect since they mediate the immunological eradication of residual tumor cells, in the context of the so called graft *versus* leukemia (GVL) effect [3]. Approaches aimed to reduce the incidence and severity of GVHD unfortunately also reduce its anti-tumor benefit [4], making the appropriate regulation of the GVHD/GVL alloreactive balance one of the milestones in the allo-SCT setting.

CD4+/CD25+/Foxp3+ regulatory T-cells (Tregs) constitute the most relevant leukocyte subtype with regulatory and suppressive functions over the immune system, playing a crucial role in autoimmunity and self-tolerance in humans [5].

After allo-SCT, there is a physiological expansion of Tregs, which are involved in the allotolerance-alloreactivity balance between donor and recipient [6,7], by suppression of antigen specific T cell responses [8]. Increased numbers of functional Tregs are known to lead to GVHD mitigation [9–12], an effect that is not necessarily associated with a decrease in the anti-tumor activity (GVL) of the allogeneic graft. However, this is still an open issue, since some authors have described attenuation of GVHD together with preservation of GVL mediated by Tregs [13,14], while others reported increased incidence of relapse in such cases [15].

Donor *versus* recipient immune reactions are also influenced by polymorphisms in certain genes coding for antigen-presenting molecules, antigen receptors, immune mediators or cellular proliferation molecules, which contribute to the development of complications after allo-SCT [16,17].

The *FOXP3* gene, located on the X chromosome (Xp11.23), which mediates the development and functional activity of Tregs [18], encodes a forkhead/winged helix transcription factor. In fact, upregulation of *FOXP3* expression is required for Treg development. Interestingly, several studies have found an association between *FOXP3* gene polymorphisms and autoimmune diseases, such as systemic lupus erythematosus [19] or preeclampsia [20]. A functional (GT)_n_ microsatellite polymorphism in a region with promoter/enhancer activity has been reported to influence *FOXP3* gene expression [21]. The presence (homo- or heterozygous females and homozygous males) of short alleles (with 15 or less microsatellite repeats; ≤(GT)_15_) is associated with a higher expression of *FOXP3*, and probably with an increase of regulatory T cell activity [22]. A number of studies have analyzed the association between this polymorphism in the promoter of the *FOXP3* gene and the development of auto- or alloimmune conditions [21,22]. Although some of them reported negative results [23–25], other showed a positive association between this polymorphism and an increased susceptibility to type1 diabetes [21] or graft rejection in renal transplant recipients [22]. Within this scenario, the (GT)_n_ polymorphism in the *FOXP3* gene might play a role in the development of certain complications after SCT, but the impact of this polymorphism in the outcome of allo-SCT has not been analyzed.

In this context, our objective was to analyze the impact of donor (GT)_n_ polymorphism in the promoter/enhancer of the *FOXP3* gene on the development of complications and ultimately on the success of conventional HLA-identical SCT.

## Patients and Methods

This retrospective study includes 252 patients with hematological malignancies, treated with myeloablative HLA-identical peripheral blood SCT (Table 1), from which donor and recipient DNA samples were available for genotyping from the DNA bank of the Spanish Group for Stem Cell Transplantation (GETH). The present study was approved by the “Area 1 Clinical Research Ethics Committee (CEIC-A1)” and therapeutic approaches, sampling and diagnostic procedures were performed after written informed consent. Diagnosis, classification and grading of GVHD were made by clinical criteria and confirmed when necessary by pathological examination of histological samples from gut, skin, liver or lung, according to international consensus criteria [26]. The median follow-up time for the cohort was 28.8 months (range 0.5–120.4).

**Table 1:** Patients, SCT features and complications developed post-SCT in the patients included in the present study.

		All patients
		n (%)
**N**		252
**Age**	Median (range)	38 (19–67)
**Patient Sex**	Male	140 (55.6)
	Female	112 (44.4)
**Donor Sex**	Male	153 (60.7)
	Female	99 (39.3)
**Donor/Recipient sex**	Female D to male R	73 (29)
**Disease**	ALL	61 (24.2)
	AML	92 (36.5)
	MDS	18 (7.1)
	MM	3 (1.2)
	Lymphoma	24 (9.5)
	Other (CML, AA, etc.)	54 (21.4)
**Disease Status at SCT** (1)	CR	155 (68.6)
	non CR	71 (31.4)
**Conditioning**	TBI	109 (43.3)
	non TBI	143 (56.7)
**Acute GVHD** (2)	Grade II-IV	79 (33.5)
	Grade III-IV	27 (11.6)
**Chronic GVHD** (3)	Any grade	105 (52.5)
	Extensive	55 (27.5)
**Relapse** (4)	Incidence	71 (31.7)
**Exitus** (5)	Total	88 (34.9)
	Relapse	37 (47.4)
	Infectious	17 (21.8)
	GVHD	16 (20.5)
	Otros	8 (10.3)
**Median OS (months) of uncensored patients (range)**		45.5 (5.7–122)

D:donor; R:recipient; ALL: acute lymphoblastic leukemia; AML: acute myeloid leukemia; MDS: myelodysplastic syndrome; MM: multiple myeloma; CML: chronic myeloid leukemia AA: aplastic anemia CR: complete remision; TBI: total body irradiation.

(1) Unknown in 26 patients. GVHD: graft versus host disease; OS: overall survival.

(2) Patients at risk (alive and in complete remission) of developing: grade II-IV aGvHD n = 236. Patients censored n = 16 (11 relapsed and 5 dead); grade III-IV aGvHD III-IV n = 232. Patients censored n = 20 (13 relapsed and 7 dead).

(3) Patients at risk: cGvHD n = 200. Patients censored n = 52 (25 relapsed and 27 dead).

(4) Patients at risk: relapse n = 224. Patients censored n = 28 (relapsed in complete remission first year).

(5) Unknown n = 10

### Genotyping of the (GT)_n_ microsatellite polymorphism

Donor and recipient genomic DNA was purified from EDTA anticoagulated peripheral blood before allo-SCT. Genotyping of the (GT)_n_ microsatellite polymorphism in the *FOXP3* gene was performed by a fluorescence-based short tandem repeat-polymerase chain reaction (STR-PCR) method (GeneAmp 7900; Applied Biosystems) and sized by capillary electrophoresis (POP7—ABI PRISM 3130 *xL* Genetic Analyzer; Applied Biosystems) and fragment analysis (GeneMapper 4.0 Software; Applied Biosystems) as previously described [21]. *FOXP3* alleles were divided in two groups: short alleles (with 15 or less microsatellite repeats; ≤(GT)_15_) and long alleles (with 16 or more microsatellite repeats; ≥(GT)_16_) [22]. Hemizygous individuals were included in their respective homozygous genotype group [27]. As suggested by Engela et al. [22], short/long heterozygous females were included in the short allele group.

### Functional effect of the (GT)_n_ microsatellite polymorphism

Luciferase assays were performed in order to determine the influence of the number of (GT)n microsatellite repeats in the promoter/enhancer on the expression of the *FOXP3* gene. Promoter activity was evaluated through the firefly luciferase activity driven by the inserted fragments upstream of the firefly luciferase gene [21]. A fragment of approximately 500 bp was amplified by PCR using the following forward: 5’-AAGGTACCGCCTCCTCACTAGCCCCACT-3’ and reverse: 5’-TTGAGCTCAAGGGCAGGCTGCGTAGACAA-3’ primers. *KpnI* and *SacI* restriction enzyme sites were introduced into each primer. PCR was carried out using Phusion DNA polymerase (Thermo Scientific Massachusetts, USA). PCR products were purified, digested by *KpnI* and *SacI* restriction enzymes at 37°C for 2 h, and incorporated into the *KpnI* and *SacI* sites of the luciferase-reporter plasmid, pGL3-Basic Vector. Five hemi- or homozygous (GT)_15_ and 5 (GT)_16_ healthy individuals were selected for this experiment. All the constructs with alleles (GT)_15_ or (GT)_16_ were verified by sequencing the inserts and flanking regions of the plasmids. HeLa cells were cultured in 1ml of DMEM medium supplemented with glutamine, antibiotics and 10% fetal calf serum under a 95% humidified air containing 5% CO_2_. HeLa cells were suspended at 7.5x10^3^ cells/ml and seeded into each of six-well plates. One day later, 1 ug (0.5 ug/well) of each plasmid construct and 0.5 ug (0.25 ug/well) of Renilla luciferase control vector (pRL-TK Renilla) were introduced to HeLa cells by the lipofection method, using Lipofectamine plus (Gibco BRL, Gaithersburg, USA). Twenty-four hours later, firefly and Renilla luciferase activities were measured using the Dual-Luciferase Reporter Assay System (Promega). Relative luciferase activity was calculated as the ratio of firefly to Renilla luciferase activity. Three different transfections were performed for each construct and each experiment was repeated three times.

### Statistical analysis

For statistical analysis, quantitative variables were expressed as median or mean and range, while qualitative variables were expressed as frequency and percentage. Testing for normality was performed with the Kolmogorov–Smirnov test. Univariate and multivariate regression analysis was performed using logistic regression (OR). For multivariate analyses, the p values were two sided and the outcomes were considered to be significant for p<0.05. Estimates of grade III-IV acute GVHD and relapse were calculated using cumulative incidence (CI) rates. Overall survival (OS) and event free survival (EFS) was calculated using the Kaplan-Meier method. Survival curves for different groups were compared by the log-rank test. Overall statistical analyses were performed using SPSS v18 for Windows (SPSS Inc., Chicago, IL, USA) and CI rates were performed by R Statistical Software ver. 2.15.0. The Hardy-Weinberg equilibrium was tested using contingency tables and Pearson's *χ* ^2^ test with SNPator software.

## Results and Discussion

Several studies have established the regulatory and suppressive functions of Tregs, mediated by *FOXP3* gene expression, over the immune system including autoimmunity and self-tolerance [5,6]. Moreover, in the allo-SCT setting Tregs are known to modulate the allotolerance-alloreactivity balance between donor and recipient [7], mitigating GVHD [9–12] while probably preserving the anti-tumor effect (GVL) of the donor graft [13].

**Fig 1:**
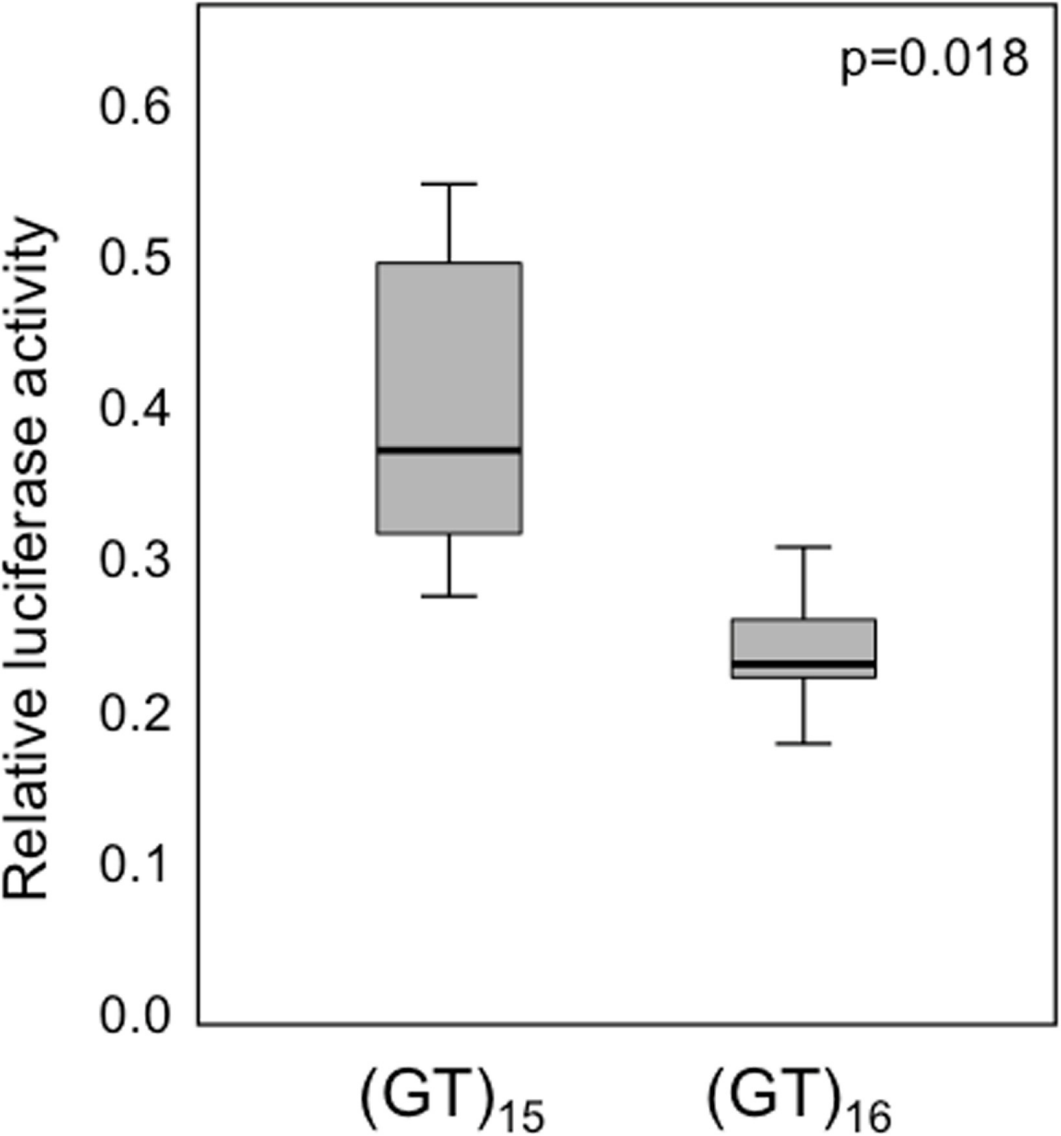
Results of the luciferase assays performed to test influence of the number of repeats in the (GT)_n_ microsatellite polymorphism on the expression of the FOXP3 gene. The (GT)_15_ allele produces significantly higher expression of the FOXP3 gene than the (GT)_16_ allele.

Polymorphisms in certain genes have shown to be implicated in the development of complications after allo-SCT [16,17]. In this context, our aim was to analyze the influence of the (GT)_n_ polymorphism in the *FOXP3* gene in the success of allo-SCT. Short alleles for such polymorphism have been shown to promote higher *FOXP3* expression and hypothetically an increase of regulatory T cell activity [21]. In our hands, luciferase assays performed to test influence of the number of repeats in the (GT)_n_ microsatellite polymorphism on the expression of the FOXP3 gene showed that the (GT)_15_ allele produces significantly higher expression of the FOXP3 gene than the (GT)_16_ allele (Fig 1).

Genotyping for the (GT)_n_ microsatellite polymorphism was carried out in a cohort of 252 unselected myeloablative HLA-identical allo-SCT patients and donors. Allelic and genotypic frequencies observed were similar to those previously reported (S1 Table) [25]. Results were in accordance with the Hardy-Weinberg equilibrium (p = 0.58).

**Fig 2:**
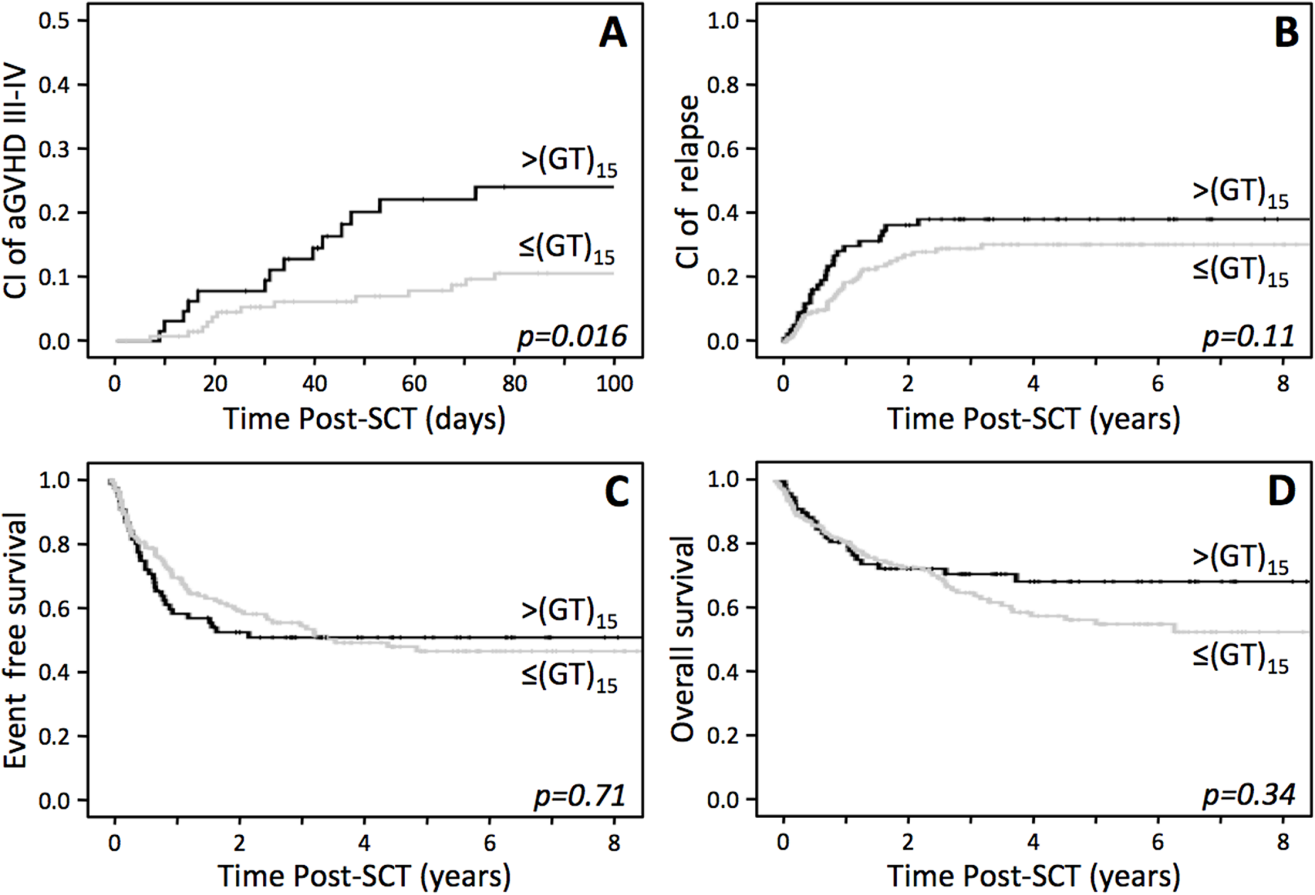
Influence of the genotype of the donor for the polymorphism (GT)_n_ in the promoter/enhancer of *FOXP3* on the outcome of allo-SCT. **(A)** Cumulative incidence of grade III-IV GVHD. **(B)** Cumulative incidence of relapse. **(C-D)** Kaplan-Meier curves of event free survival (B) and overall survival (C).

The genotype of the recipient for the (GT)_n_ polymorphism did not influence SCT outcome (data not shown) supporting previous observations [11] that showed that the amount of Tregs in the donors influenced SCT outcomes. Indeed, as expected from the reported observations mentioned above, the presence of short alleles in the donor was associated with a lower incidence of grade III-IV acute GVHD with statistically significant association (OR = 0.36, CI = 0.16–0.82, p = 0.016; Table 2). After multivariate analysis introducing all potentially confounding variables (Table 3), the presence of short alleles in the donor remained as an independent protective factor for the development of grade III-IV acute GVHD (OR = 0.26, CI = 0.08–0.82, p = 0.02). Total body irradiation (TBI) used within the conditioning regimen for ALL patients has shown to be associated with the development of GVHD [28] and these two factors (ALL and TBI) are also identified in the present study (Table 3). Additionally, CI of grade III-IV acute GVHD was significantly lower in patients transplanted from short allele donors (CI 100 days 8.3% *vs*. 20.7%, p = 0.016, Fig 2A). On the other hand, no significant association was observed for moderate-severe chronic GVHD (OR = 1.1, CI = 0.56–2.19, p = 0.86; Table 2).

**Table 2:** Univariate analysis of the association between the presence of FOXP3 short alleles in the donor and the development of post-SCT complications.

	Univariate	
	OR (95% CI)	p-value
**Grade II-IV aGVHD**	0.67 (0.37–1.19)	0.174
**Grade III-IV aGVHD**	0.36 (0.16–0.82)	**0.016** *
**cGVHD**	0.84 (0.46–1.54)	0.58
**Extensive cGVHD**	1.1 (0.56–2.19)	0.76
**Relapse**	0.62 (0.35–1.1)	0.1
**Mortality**		
**Overall**	1.28 (0.72–2.25)	0.4
**Relapse**	0.59 (0.22–1.59)	0.3
**GVHD**	0.63 (0.19–2)	0.43
**Infections** (1)	3.93 (0.82–1.89)	0.087

aGVHD: acute graft versus host disease; cGVHD: chronic GVHD.

(1)Seventeen patients died from infections. Data available for 12 patients, all of them due to bacterial infections, 5 died during the first year (range 2–10 months) and 7 died beyond the first year (range 15–48 months).

* p<0.05

**Table 3:** Univariate and multivariate analysis for variables potentially associated with the development of grade III-IV aGvHD.

	Univariate		Multivariate	
	OR (95% CI)	p-value	OR (95% CI)	p-value
**FOXP3 short allele donor**	0.36 (0.16–0.82)	**p = 0.016** *	0.26 (0.08–0.82)	**p = 0.021** *
**FOXP3 short allele recipient**	1.1 (0.47–2.6)	p = 0.81	1.74 (0.54–5.6)	p = 0.35
**Patient age**	1.12 (0.5–2.5)	p = 0.78	1.4 (0.5–3.95)	p = 0.52
**Donor sex**	1.05 (0.47–2.39)	p = 0.9	2.32 (0.84–6.41)	p = 0.11
**Recipient sex**	1.4 (0.63–3.1)	p = 0.4	1.36 (0.41–4.49)	p = 0.62
**Female donor/male recipient**	1.44 (0.56–3.77)	p = 0.44	0.99 (0.27–3.65)	p = 0.99
**Disease**				
ALL	2 (0.87–4.71)	p = 0.1	4.55 (1.09–18.94)	**p = 0.037** *
AML	0.35 (0.13–0.97)	p = 0.043	0.43 (0.1-.079)	p = 0.25
MDS	1.17 (0.25–5.52)	p = 0.83	0.89 (0.15–5.49)	p = 0.9
MM	1		1	
Lymphoma	1.37 (0.38–5.04)	p = 0.63	1.54 (0.27–8.85)	p = 0.63
Other	1.34 (0.49–3.11)	p = 0.65	1.14 (0.45–2.91)	p = 0.78
**Disease status at allo-SCT**	1.45 (0.6–3.5)	p = 0.42	1.62 (0.59–4.49)	p = 0.35
**TBI in the conditioning**	2.24 (0.91–5.52)	p = 0.081	6.45 (1.79–23.16)	**p = 0.04** *

ALL: acute lymphoblastic leukemia; AML acute myeloid leukemia; MDS: myelodysplastic syndromes; MM: multiple myeloma; CML: chronic myeloid leukemia; SCT: stem cell transplantation; TBI: total body irradiation.

* p<0.05

Treg cell counts were not measured prospectively and the registry-based nature of the present study does not allow such information to be obtained. However, T cell (CD3/CD4/CD8) reconstitution data were available for a subset of patients (S1 Fig). Although no statistical differences were observed, T cells, mainly CD8 + cells, were lower at day +60 in (GT)_15_ patients. Interestingly, differences are lost later on after transplant (day +90). The effect of the polymorphism on the immune response after transplantation might be restricted to the early post-SCT period and have less influence in later post-SCT phases. This fact that could account for the absence of relationship between the polymorphism in *FOXP3* and the development of cGVHD.

Interestingly, increased numbers of functional Tregs are not necessarily associated with a decrease in the anti-tumor activity (GVL) [13]. In fact, the presence of short alleles in the donor was not associated with a higher risk of relapse (OR = 0.62, CI = 0.35–1.1, p = 0.1; Table 2) in this cohort. Moreover, CI of relapse was not different between patients transplanted from short or long allele donors (CI at 2 years 33.3% *vs*. 25%, p = 0.11, Fig 2B). Therefore, the presence of short alleles in the donor did not affect GVL in the present series.

Finally, an impact of the (GT)_n_ polymorphism on the risk of death was not observed (OR = 1.28, CI = 0.72–2.25, p = 0.4, Table 2). Moreover, no statistically significant association was found in terms of EFS and OS (EFS, median time 54.1 months *vs* not reached, p = 0.71 and OS, 113.2 vs 110.6 months, p = 0.344; Fig 2C and 2D). Noteworthy, patients transplanted from short allele donors showed a trend to a higher incidence of mortality derived from infectious complications (OR = 3.93, CI = 0.82–18.9, p = 0.087, Table 2). In fact, the suppressive function of regulatory T cells has been related to a higher incidence of infections [29].

Of note, the association described above between the presence of short alleles and the development of grade III-IV aGVHD is also observed when transplants from male and female donors are analysed separately (S2 Table).

Summarizing, our results are in agreement with previous observations since donors harboring short alleles, which have been associated with higher *FOXP3* gene expression and greater production of Tregs [21] are less alloreactive and, therefore, their recipients develop less acute GVHD. Interestingly, these patients do not show higher relapse rates since Tregs are thought to reduce acute GVHD probably without affecting the beneficial GVL effect [13]. Greater numbers of Tregs in GVHD target tissues–accounting for the amelioration of GVHD–than in the BM of transplanted patients–allowing effective GVL responses to be mounted–has been postulated as a possible explanation for this observation [14]. Further prospective studies correlating the genotype analysis with Treg frequency would be of interest to confirm these data.

To the best of our knowledge, this is the first report of the implication of (GT)_n_ microsatellite polymorphism of the promoter/enhancer region of *FOXP3* gene in the outcome of allo-SCT. Analysis of this polymorphism can help in appropriate donor selection and, more importantly, drive a tailored management of patients submitted to allo-SCT.

## Supporting Information

S1 Fig Box plot showing CD3+, CD4+ and CD8+ cell counts as determined by flow cytometry in erythrocyte-lysed whole PB samples obtained at days +60 and +90 after stem cell transplantation in patients transplanted from (GT)_15_ (white boxes; n = 37) or (GT)_16_ (grey boxes, n = 29) donors. Although not significant differences are observed, cell counts (mostly CD3+ and CD8+ cells) at day +60 appear higher in patients transplanted from (GT)_16_ donors. Such differences are lost when patients are studied at day +90.
(TIF)

S1 Table Genotypes for the (GT)n polymorphism in the FOXP3 gene observed in the cohort of patients and donors included in this study. (XLS)

S2 Table Univariate analysis of the association between the presence of FOXP3 short alleles in female or male donors and the development of post-SCT complications. aGVHD: acute graft versus host disease; cGVHD: chronic GVHD.
(XLS)
