# Supplementary figures and images for: The Genotype of the Donor for the (GT)n Polymorphism in the Promoter/Enhancer of FOXP3 Is Associated with the Development of Severe Acute GVHD but Does Not Affect the GVL Effect after Myeloablative HLA-Identical Allogeneic Stem Cell Transplantation
Source: PLoS One. 2015 Oct 16;10(10):e0140454. doi: 10.1371/journal.pone.0140454 (PMC4608671; doi:10.1371/journal.pone.0140454)

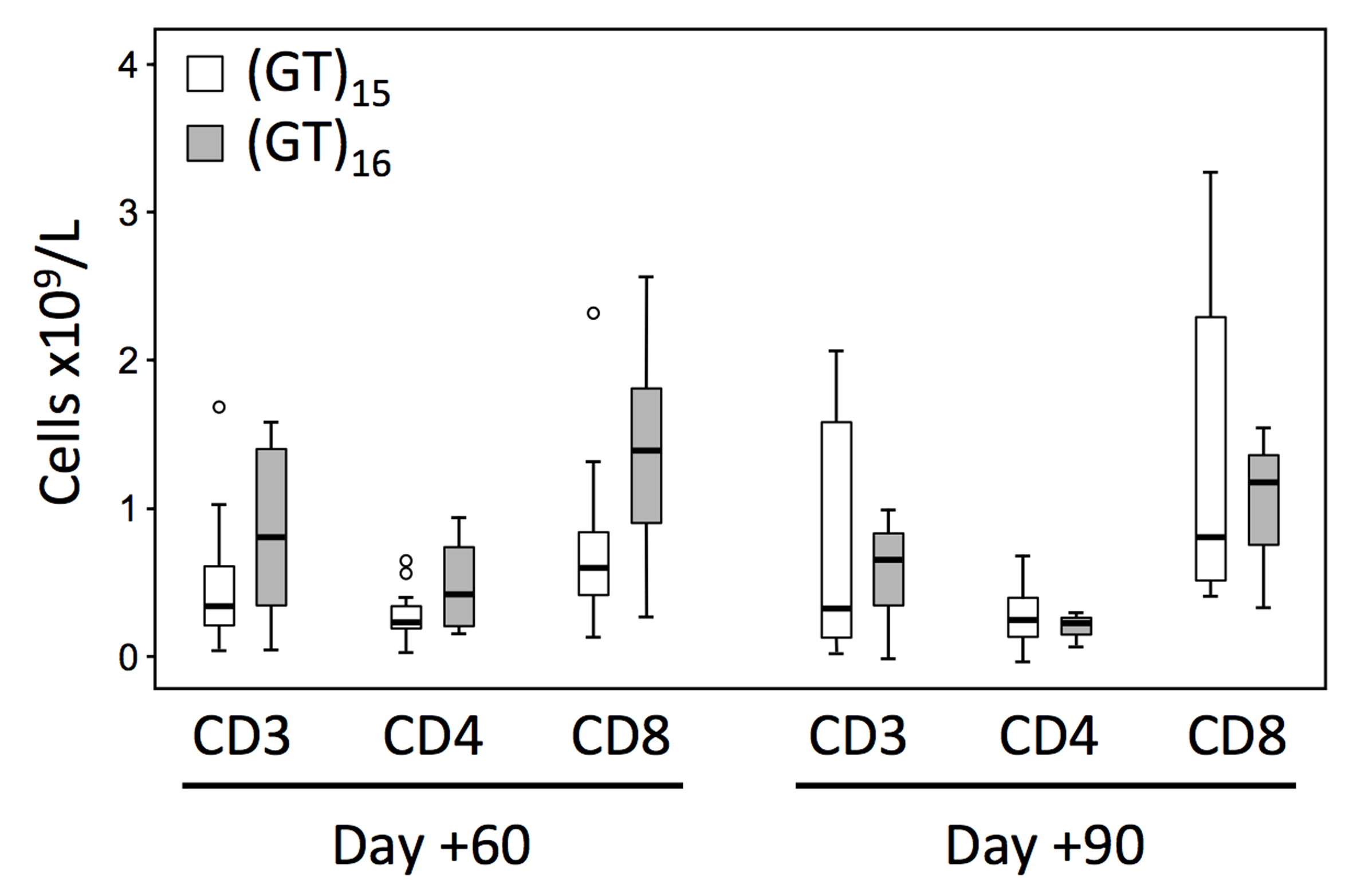

Supplement: S1 Fig — Although not significant differences are observed, cell counts (mostly CD3+ and CD8+ cells) at day +60 appear higher in patients transplanted from (GT)16 donors. Such differences are lost when patients are studied at day +90. (TIF) [file pone.0140454.s001.tif]
